# Exploring the Possible Cause of the Dramatic Increase in Measles Mortality During the 2015–2016 Mongolian Outbreak

**DOI:** 10.1093/infdis/jiaa084

**Published:** 2020-02-27

**Authors:** Lien Anh Ha Do, Naranzul Tsedenbal, Claire von Mollendorf, Tuya Mungun, Darmaa Bardach, Kim Mulholland

**Affiliations:** 1 New Vaccines Group, Murdoch Children’s Research Institute, Melbourne, Australia; 2 Department of Paediatrics, The University of Melbourne, Melbourne, Australia; 3 National Center of Communicable Diseases, Ulaanbaatar, Mongolia; 4 London School of Hygiene and Tropical Medicine, London, United Kingdom

**Keywords:** measles virus, measles mortality, respiratory syncytial virus, influenza, immunosuppression

To the Editor—Measles case fatality rates vary greatly between outbreaks for reasons that are not well understood [1]. In Mongolia, measles returned dramatically as a bimodal epidemic during 2015 and 2016 [2, 3]. The infant measles mortality rate during the 2016 wave, was 10 times higher than during the 2015 wave [2, 3]. Coinfection with influenza B was suggested by Lee and colleagues to be the likely cause of the increase [2, 3], yet from 132 measles deaths only 6 lung tissue samples were tested for pathogens, of which 2 were positive for influenza B [2, 3].

Since April 2015, we have established ongoing childhood pneumonia surveillance in 4 of the 9 districts of Ulaanbaatar, Mongolia, to evaluate the impact of introducing pneumococcal conjugate vaccine [4]. From April 2015 to September 2016, we screened 1431 children <2 years of age who were admitted with severe pneumonia for respiratory syncytial virus (RSV) and influenza, and we made 2 surprising observations: first, an apparently stronger coincidence of measles deaths with the peak of RSV detection among children with pneumonia, and second, an intriguingly dramatic increase in all pneumonia admissions and severe pneumonia cases associated with RSV, but not with influenza A or B, in the year after the measles virus (MeV) outbreak (Figure 1). 

**Figure 1.**
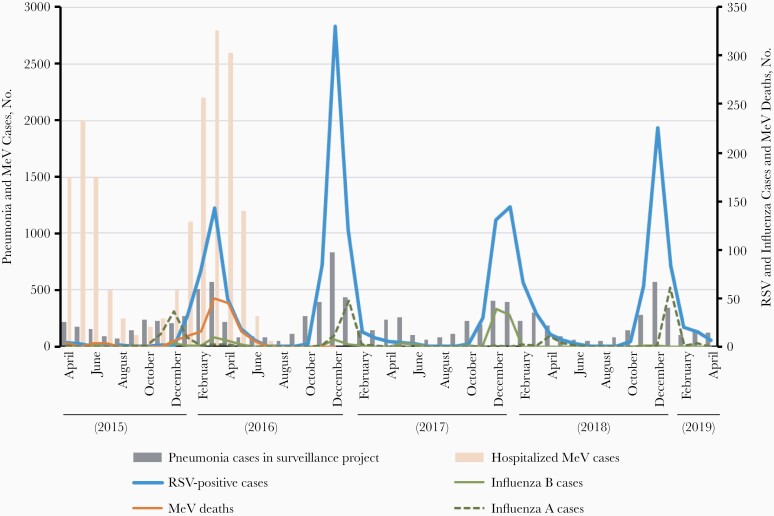
Measles cases and measles deaths during pneumonia, respiratory syncytial virus (RSV) and influenza surveillance (measles data from Orsoo et al [2]).

These observations and those of Lee and colleagues [3] could be explained by a phenomenon known as “measles amnesia.” This refers to immunosuppression secondary to MeV infection increasing host susceptibility to other nonmeasles pathogens, both during the acute MeV infection phase and during the months after the MeV infection has resolved. The immunosuppression is due to the depletion of preexisting memory T and B cells specific to nonmeasles pathogens, as demonstrated in a macaque model [5]. 

In a later study, Mina et al [6] were able to profile the immune memory antibody repertoire against a range of viral and bacterial epitopes in plasma collected before and after measles infection in a cohort of 77 unvaccinated children in the Netherlands. After mild and severe MeV infections, children lost a median of 20% and 40%, respectively of their total preexisting pathogen-specific antibody repertoire. This loss varied among children and specific pathogens. It was observed across nearly all subjects for their antibody repertoires for *Streptococcus pneumoniae*, influenza B, enterovirus, rhinovirus and RSV. A significant reduction in the avidity of antibody binding to the important palivizumab binding site of RSV was also observed in 22 of the 77 children. 

These findings were confirmed again by similar findings from the macaque model, where MeV infection led to variable reductions in RSV and influenza B antibody repertoires of 45% and 60%, respectively [6]. This loss of antibody repertoire in macaques persisted for at least 5 months, and epidemiological evidence in humans suggests there is increased susceptibility to deaths from nonmeasles infectious disease for up to 5 years after a measles outbreak [6]. In applying these findings to the Mongolian outbreak, it seems that the excess measles mortality rate may be due to RSV or influenza B infection leading to a fatal outcome among children recovering from measles. This may also partly explain the excess of RSV-associated pneumonia admissions the following winter.

Another side of the interplay between MeV and RSV is the potential heterologous protection between the 2 viruses, because they belong to the same Paramyxoviridae family. T-cell cross reactive responses between RSV and MeV have been reported for in vitro and in vivo mice models [7]. Better immunoglobulin G responses to measles vaccine were shown in patients previously exposed to RSV than in those never infected by RSV [8]. In addition, MeV vaccine has been shown to have unexpected beneficial (nonspecific) effects on general morbidity and mortality rates associated with infectious diseases, including RSV infection specifically [9]. 

In conclusion, measles mortality rates vary greatly between epidemics and regions, but few studies have interrogated the causes of measles-associated deaths, mainly because of complex logistic, administrative and research capacity limitations during MeV epidemics. The potential link between RSV and MeV warrants further investigation with the goal of better controlling both diseases.

## Notes

***Acknowledgments.*** This study would not have been possible without the ongoing dedication and support from clinicians at the 4 district hospitals (Chingeltei, Bayanzurkh, Songinokhairkhan, and Sukhbaatar) and the tertiary Maternal and Child Health Hospital, in Ulaanbaatar, Mongolia. We also thank the Mongolian Ministry of Health, the National Centre for Communicable Diseases, especially the pneumococcal conjugate vaccine study team and the virology laboratory, and the local World Health Organization team in Ulaanbaatar for their support.

***Disclaimer.*** The funder had no role in study design, data collection and analysis, the decision to publish, or the preparation of the manuscript.

***Financial support.*** This work was supported by the Bill & Melinda Gates Foundation (grant OPP1169299).

***Potential conflicts of interest.*** All authors: No reported conflicts. All authors have submitted the ICMJE Form for Disclosure of Potential Conflicts of Interest. Conflicts that the editors consider relevant to the content of the manuscript have been disclosed.
